# National Bariatric Surgery Registries: an International Comparison

**DOI:** 10.1007/s11695-021-05359-0

**Published:** 2021-03-30

**Authors:** Erman O. Akpinar, Perla J. Marang- van de Mheen, Simon W. Nienhuijs, Jan Willem M. Greve, Ronald S. L. Liem

**Affiliations:** 1 Department of Surgery, Maastricht University Medical Centre, Maastricht, Netherlands; 2 Scientific Bureau, Dutch Institute for Clinical Auditing, Leiden, Netherlands; 3 Department of Biomedical Data Sciences, Medical Decision Making, Leiden University Medical Centre, Leiden, Netherlands; 4 Department of Surgery, Catharina Hospital, Eindhoven, Netherlands; 5 Department of Surgery, Zuyderland Medical Centre, Heerlen, Netherlands; 6 Dutch Obesity Clinic South, Heerlen, Netherlands; 7 Department of Surgery, Groene Hart Hospital, Gouda, Netherlands; 8 Dutch Obesity Clinic, The Hague, Netherlands

**Keywords:** Bariatric surgery, Obesity, IFSO, Population-based, National bariatric surgery registries, Common data elements

## Abstract

**Introduction:**

Pooling population-based data from all national bariatric registries may provide international real-world evidence for outcomes that will help establish a universal standard of care, provided that the same variables and definitions are used. Therefore, this study aims to assess the concordance of variables across national registries to identify which outcomes can be used for international collaborations.

**Methods:**

All 18 countries with a national bariatric registry who contributed to The International Federation for the Surgery of Obesity and Metabolic Disorders (IFSO) Global Registry report 2019 were requested to share their data dictionary by email. The primary outcome was the percentage of perfect agreement for variables by domain: patient, prior bariatric history, screening, operation, complication, and follow-up. Perfect agreement was defined as 100% concordance, meaning that the variable was registered with the same definition across all registries. Secondary outcomes were defined as variables having “substantial agreement” (75–99.9%) and “moderate agreement” (50–74.9%) across registries.

**Results:**

Eleven registries responded and had a total of 2585 recorded variables that were grouped into 250 variables measuring the same concept. A total of 25 (10%) variables have a perfect agreement across all domains: 3 (18.75%) for the patient domain, 0 (0.0%) for prior bariatric history, 5 (8.2%) for screening, 6 (11.8%) for operation, 5 (8.8%) for complications, and 6 (11.8%) for follow-up. Furthermore, 28 (11.2%) variables have substantial agreement and 59 (23.6%) variables have moderate agreement across registries.

**Conclusion:**

There is limited uniform agreement in variables across national bariatric registries. Further alignment and uniformity in collected variables are required to enable future international collaborations and comparison.

**Graphical abstract:**

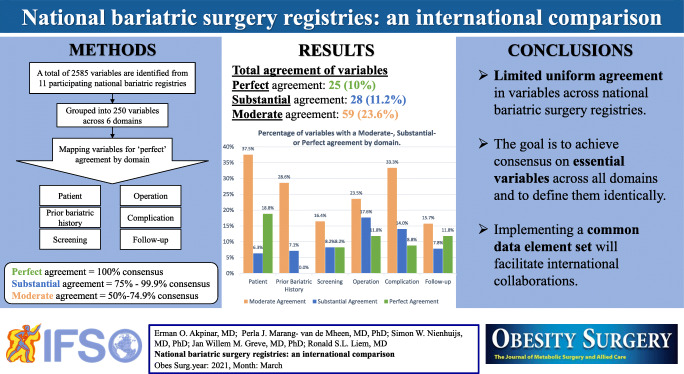

**Supplementary Information:**

The online version contains supplementary material available at 10.1007/s11695-021-05359-0.

## Introduction

National bariatric surgery registries ensure and improve the quality of care provided to the patient [1–4]. Pooling the datasets from all the national bariatric registries may provide international real-world evidence that will help establish a universal standard of care for the treatment of patients with morbid obesity [5–7].

The International Federation for the Surgery of Obesity and Metabolic Disorders (IFSO) global registry report 2019 includes a total of 833.687 operation records combining all bariatric registries [8]. The main goal of the global registry is to improve outcomes for bariatric patients. However, there is a structural lack of consistency in defining outcomes across national registries [9–11]. Most registries do not register the same variables, and even when they register the same variables, it only has a similar “overall concept”, rather than the same definition. For example, the same overall concept being measured with “severe postoperative complications” may contain categories such as bleeding or leakage but is defined differently in other registries where additional categories such as obstruction or stricture are also included [12]. A “common language” in gathering and defining variables is required to address the issues mentioned above, resulting in common data elements (CDEs) and eventually leading to standardized outcome reporting[13], but the extent of inconsistency across registries is currently unknown.

Therefore, this study aimed to compare the degree of concordance in variables between national bariatric registries and to discuss the need for further alignment and uniformity in collecting variables for international collaborative and comparative studies.

## Materials and Methods

### Study Design

The IFSO Registry Committee requested all 18 countries with an established national bariatric surgery registry in 2019 by email to share their data dictionary for this study [11]. The Committee requested countries that did not include the definition of variables in their data dictionary to send a separate explanatory text guiding consistent data entry, e.g., appearing as part of hover prompts containing the definition of variables. A reminder was sent by email after 2–4 weeks to ensure a high participation rate.

### Review of Variables

Registries differ in whether they use one or more variable(s) to measure the same overall concept. For example, the variable “diabetes mellitus diagnosis” with the categories (1) “no”, (2) “yes”, and (3) “yes, with medication” can be followed by a second variable “details of the treatment” with the categories (1) oral hypoglycemics, (2) insulin treatment, and (3) injectable other than insulin. These two variables measure the same overall concept as one variable with the categories: (1) no indication of diabetes, (2) pre-diabetes, (3) oral hypoglycemics, and (4) insulin treatment. Variables are registry specific and thus challenging to compare 1 on 1. To assess the degree of concordance for this study, variables were first grouped in variables that measure the same overall concept and then categorized into the following six domains: patient characteristics, prior bariatric history, screening, operation, complication, and follow-up. These domains are based on the chronological order of the care pathway that appears in most national registries. For each individual registry, their variables were mapped against the total list of grouped variables within the different domains. During the mapping of individual registries, the following main points were taken into account: whether the content of the variable(s) occur in the registry (registered/not registered) and whether the variable(s) have a matching definition. Upon receiving the data dictionaries, a medical doctor (EA) listed all variables from all participating registries. Then, two reviewers, a medical doctor (EA), and expert bariatric surgeon (RL) had several meetings as part of the mapping process to review and discuss the assignment of variables in the different domains. A third independent expert bariatric surgeon (SN) was available to discuss until consensus was reached in case of disagreement. When the definition of variables was not available, the variables were reviewed to the best of our knowledge with the provided documents at hand.

### Defining Variables

Continuous variables, e.g., “weight”, and categorical variables, e.g., “diabetes mellitus diagnosis”, containing the categories “yes” or “no” were considered a match if they had the same definition. Categorical variables were also considered a match if they could be mapped to a higher-level aggregated category, e.g., “postoperative myocardial infarct” or “postoperative dysrhythmia” matches the aggregated category “cardiac complications”.

### Outcome parameters

The primary outcome was the percentage of “perfect agreement” across registries for variables by domain. Perfect agreement was defined as a 100% concordance for variables across all registries, meaning that the variable is recorded in all registries with the same definition or matches the mapping of a categorical variable with the same aggregated category. Secondary outcomes were “75–99.9%” concordance defined as “substantial agreement” and “50–74.9%” concordance defined as “moderate agreement”.

## Results

### Participating Registries

Eleven out of 18 national bariatric surgery registries responded and agreed to participate in the study, as shown in Table 1. The 18 national registries together comprise a total of 735.881 patients, from which the 11 participating registries included *n* = 554.599 (75.4%) patients undergoing bariatric surgery according to the IFSO Global Registry report 2019 [8]. Registries with definitions available for part of the variables were from Brazil, Kuwait, Mexico, Russia, Turkey, and the UK.

**Table 1:** Participating national bariatric surgery registries (in alphabetical order)

Number	Country	Registration name	Participating	Country-specific definition of variables
1	Australia/New Zealand^a^	ANZMOSS	Yes	Yes
2	Austria	OGA	Yes	No
3	Belgium	BeSOMS	No	-
4	Brazil	SBCBM	Yes	Yes (partially)
5	Egypt	ESBS	No	-
6	France	SOFFCO.MM	No	-
7	India	OSSI	No	-
8	Israel	ISMBS	No	-
9	Italy	SICOB	No	-
10	Japan	JSSO	No	-
11	Kuwait	KLOSS	Yes	Yes (partially)
12	Netherlands	DATO	Yes	Yes
13	Norway^b^	SOREG-N	Yes	Yes
14	Russia	BAREOREG	Yes	Yes (partially)
15	Sweden^b^	SOREG-S	Yes	Yes
16	Turkey	TOSS	Yes	Yes (partially)
17	UK	NBSR	Yes	Yes (partially)
18	USA	MBSAQIP	Yes	Yes

^a^Australia and New Zealand share an identical national registry and are therefore counted as one registry

^b^Norway and Sweden register independently and are counted as two registries, but use identical data dictionaries that are compatible when merging data

### Primary and Secondary Outcomes

**Fig. 1:**
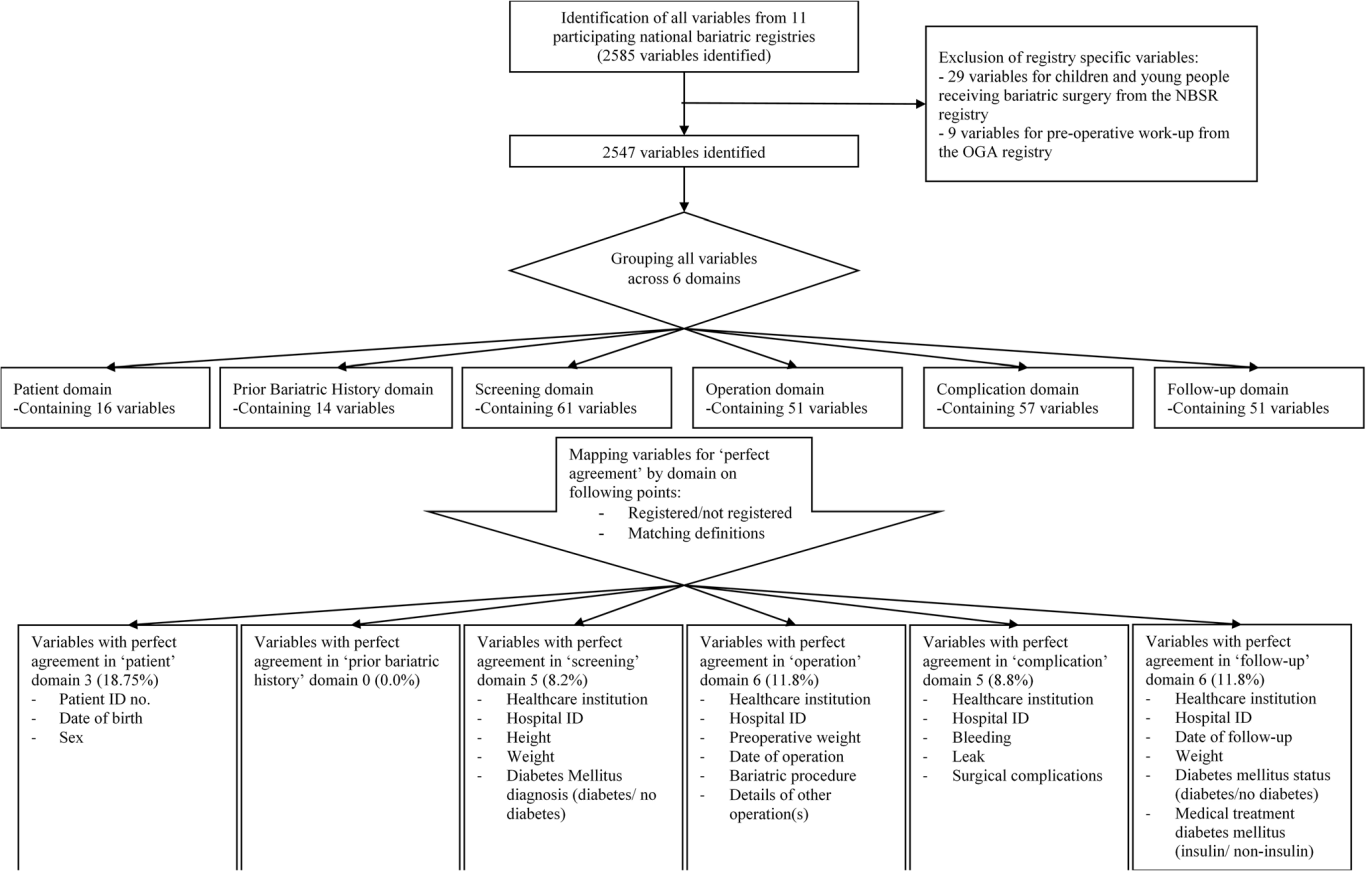
Flowchart for identifying perfect agreement in variables across 11 national bariatric registries

**Fig. 2:**
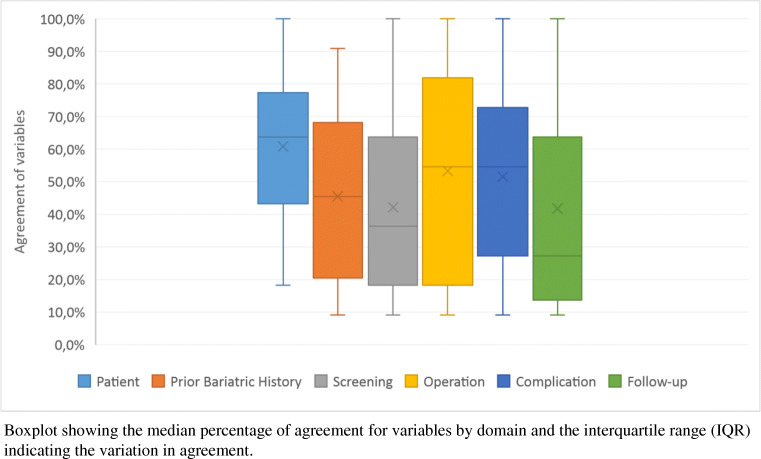
Boxplot for the median agreement rates of variables by domain

A total of 2585 variables were assessed, which were grouped into 250 variables (Supplementary Table 1) measuring the same concept across the 6 domains. From these 250 variables, 16 (6.4%) are in the patient domain, 14 (5.6%) in prior bariatric history, 61 (24.4%) in screening, 51 (20.4%) in operation, 57 (22.8%) in complication, and 51 (20.4%) in follow-up (Fig. 1). The number of variables with perfect agreement by domain was: 3 (18.75%) for the patient domain, 0 (0.0%) for prior bariatric history, 5 (8.2%) for screening, 6 (11.8%) for operation, 5 (8.8%) for complications, and 6 (11.8%) for follow-up, meaning a total of 25 (10%) variables across all domains. Perfect agreement was found for the variables “hospital ID” and “healthcare institution” that were part of the domains screening, operation, complications, and follow-up. Within the domain “complications”, perfect agreement was found for the 3 variables “postoperative bleeding”, “leak”, and “surgical complication” with the first two having identical definitions and the latter mapped to the same aggregated category. Within the follow-up domain, the 4 variables with a perfect agreement were “date of follow-up”, “weight”, “medical treatment of diabetes mellitus” with the categories (1) “insulin”, (2) “non-insulin medication”, and “diabetes mellitus status” with the categories (1) diabetes or (2) no diabetes. Figure 2 shows the median percentage of agreement for variables by domain and the interquartile range (IQR) indicating the variation in agreement rather than only looking at perfect agreement: patient 63.6% [IQR = 43.2–77.3%], prior bariatric history 45.5% [IQR = 20.5–68.2%], screening 36.4% [IQR = 18.2–63.6%], operation 54.5% [IQR = 18.2–81.8%], complication 54.5% [IQR = 27.3–72.7%], and follow-up 27.3% [IQR = 13.6–63.6%].

A summary of the variables with a “perfect”, “substantial”, and “moderate” agreement is shown in Table 2. A total of 28 (11.2%) variables have substantial agreement (75–99.9%), and a total of 59 (23.6%) variables have moderate agreement (50–74.9%) across registries. Taken together, this means that from the total of 250 variables in all registries, 138 (55.2%) variables had less than 50% agreement across registries.

**Table 2:** Summary of variables divided into perfect, substantial, and moderate agreement

	Variables	Perfect agreement 100%	Substantial agreement 75–99.9%	Moderate agreement 50–74.99%
Patient characteristics				
1.	Nationality			X
2.	Patient ID no.	X		
3.	Healthcare institution			X
4.	Hospital ID		X	
5.	Initials			X
6.	Prefix			X
7.	Surname			X
8.	Date of birth	X		
9.	Sex	X		
10	Date of death			X
Prior bariatric history				
11.	Hospital ID			X
12.	Prior metabolic or bariatric procedure		X	
13.	Prior type of gastric bypass			X
14.	Prior type of malabsorptive procedure			X
15.	Prior type of other bariatric procedures			X
Screening				
16.	Healthcare institution	X		
17.	Hospital ID	X		
18.	Date of consultancy		X	
19.	Height	X		
20.	Weight	X		
21.	Hypertension (diagnosis)		X	
22.	Diabetes mellitus (diagnosis)	X		
23.	Details diabetes mellitus		X	
24.	HbA1c (mmol/mol)			X
25.	Dyslipidemia			X
26.	GERD (diagnosis)			X
27.	OSAS (diagnosis)		X	
28.	Osteoarthritis (diagnosis)		X	
29.	Peripheral vascular disease/aneurysm aorta			X
30.	Liver disease			X
31.	Mobility			X
32.	Increased risk pulmonary Embolism			X
33.	PCOS			X
34.	Depression			X
35.	Smoking			X
Operation				
36.	Healthcare institution	X		
37.	Hospital ID	X		
38.	Preoperative weight	X		
39.	ASA classification			X
40.	Date of operation	X		
41.	Surgical procedure (primary/two-stage/revision)		X	
42.	Operative approach		X	
43.	Bariatric procedure	X		
44.	Surgeon ID			X
45.	Date of discharge			X
46.	Type of technique gastric band		X	
47.	Fixation gastric band			X
48.	Type malabsorptive		X	
49.	Type gastric bypass		X	
50.	Biliopancreatic limb length			X
51.	Alimentary limb length			X
52.	Closure Petersen’s space		X	
53	Closure hernia jejuno-jejunostomy		X	
54.	Type gastric band (brand)			X
55.	Common limb length		X	
56.	Bougie size		X	
57.	Technique of pouch excision			X
58.	Distance from pylorus			X
59.	Details of other operation(s)	X		
60.	Combined operation			X
61.	Suture material			X
62.	Ante-colic/retro-colic			X
Complication				
63.	Healthcare institution	X		
64.	Hospital ID	X		
65.	Date of complication		X	
66.	Period the complication occurred		X	
67.	Date of re-admission			X
68.	Date of discharge after re-admission			X
69.	Type of (re)intervention			X
70.	Operative approach (re)intervention			X
71.	Patient status at discharge		X	
72.	Gastrointestinal perforation		X	
73.	Bleeding	X		
74.	Splenic injury			X
75.	Source of bleeding			X
76.	Surgical complications	X		
77.	Leak	X		
78.	Post-operative complications		X	
79.	Gastric complication			X
80.	Stricture			X
81.	Electrolyte disorder			X
82.	Hepatobiliary problems			X
83	CBD stones			X
84.	Band problems			X
85.	Pouch dilatation/band slippage			X
86.	Band erosion			X
87.	Port/band infection			X
88.	Other complications (including cardiac, pulmonary and other)		X	
89.	Incisional hernia		X	
90.	Intestinal obstruction		X	
91.	Petersen’s hernia			X
92.	Malnutrition/enteral feeding			X
93.	Post-op vomiting/nausea			X
94.	Patient discharge to (home/revalidation center)			X
Follow-up				
95.	Healthcare institution	X		
96.	Hospital ID	X		
97.	Date of follow-up	X		
98.	Weight	X		
99.	Hypertension status		X	
100.	Medical treatment hypertension			X
101.	Diabetes mellitus status	X		
102.	HbA1c (mmol/mol)			X
103.	Medical treatment diabetes mellitus	X		
104.	Dyslipidemia status		X	
105.	GERD status		X	
106.	Medical treatment GERD			X
107.	OSAS status		X	
108.	Medical treatment OSAS			X
109.	Osteoarthritis			X
110.	Medical treatment osteoarthritis			X
111.	Clinical malnutrition			X
112.	Vitamins and micro-elements intake			X

*ID*, identity document; *HbA1c*, hemoglobin A1c; *GERD*, gastroesophageal reflux disease; *OSAS*, obstructive sleep apnea syndrome; *PCOS*, polycystic ovary syndrome; *ASA*, American Society of Anesthesiologists; *CBD stones*, common bile duct stones

Adolescent section of the NBSR and the pre-operative work-up section of OGA are not included in the list of common data elements due to registration-specific variables

**Fig. 3:**
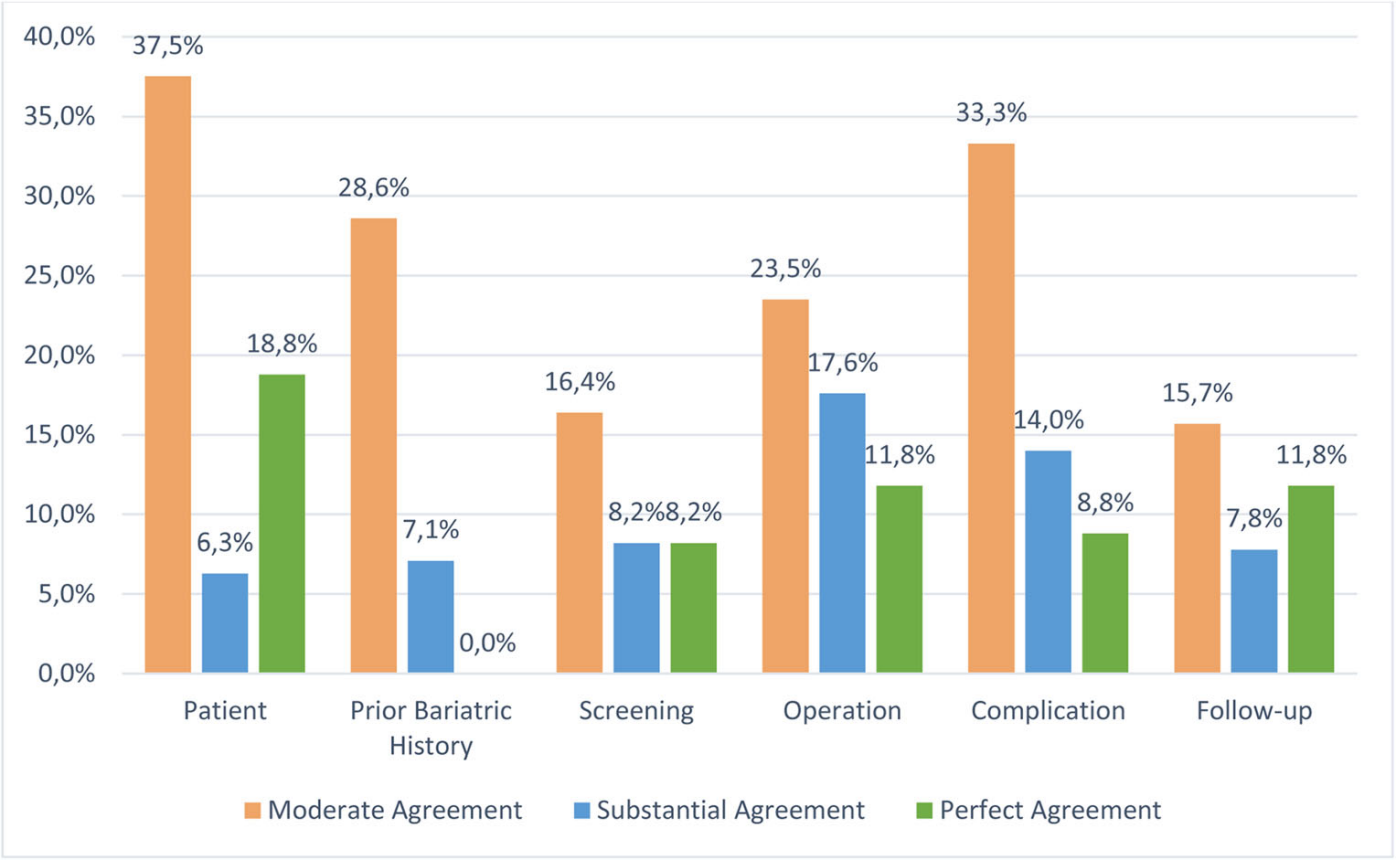
Percentage of variables with a moderate, substantial, or perfect agreement by domain. Moderate agreement is 50–74.9% consensus, substantial agreement is 75–99.9% consensus, and perfect agreement is 100% consensus

Figure 3 shows how these variables with perfect, substantial, and moderate agreement are distributed across the 6 domains. The domains, patient, operation, and complication, have 10(62.5%), 27(52.9%), and 32(56.1%) variables, respectively, with more than 50% agreement across registries.

## Discussion

The present study aimed to assess the concordance in variables across participating national bariatric surgery registries. Even though participating registries in this study include a larger number of patients (*n* = 554.599) than individual registries, data can only be pooled as part of a collaborative study if there is a common language on collected data between national registries. To our knowledge, this is the first comparison of variables between national bariatric surgery registries, showing that there is only limited “perfect agreement” of variables, suggesting that there is a need for better alignment and uniformity in collecting variables across national bariatric surgery registries.

Although this is the first comparison of variables between national registries, several limitations should be noted. First, not all national registries participated, meaning that the current agreement percentage could be an overestimation. Furthermore, not all participating registries had specific definitions for all variables available, resulting in some variables assessed to the best of our knowledge with the documents at hand. Additionally, there were registration-specific variables such as the adolescent-specific variables of the UK registration and the pre-operative work-up variables in the Austria registration, which we did not include because the individual registries were the only ones collecting them. Including these domains would have led to even more discrepancy in the agreement between registrations and further support our findings of limited concordance. Finally, this study only looked at the concordance of variables currently collected in national registries; however, this does not reflect importance, meaning that other (not yet reported) variables may be considered essential in decisions towards a bariatric common data element (CDE) set.

The BARIACT project has proposed a core outcome set including nine outcomes [14]. However, the core outcome set is developed in the UK, making it specific for the UK population rather than internationally applicable. Furthermore, it only contains outcomes and does not contain all essential variables across all domains. Our study compares on an international level and shows that perfect agreement on variables occurs across all six domains, showing the importance of variables such as patient characteristics, and operation details.

To assess the degree of concordance for this study, we grouped the variables that measure the same overall concept. Brethauer et al. recommend using standardized outcome reporting and encounter challenges when reporting, e.g., “complete diabetes remission” [15]. Whereas the ASMBS recommends a lower HbA1c level < 6% without the use of glucose-lowering medication [15], the International Diabetes Federation (IDF) target is HbA1c < 7% with or without medication [16, 17]. Our study also encountered these challenges, showing that there is a need to not only register these variables but also to define them identically.

## Future Perspectives

This study provides an overview of the currently collected variables from participating countries, and it could serve as a stepping stone in developing a CDE set on a broader scale. IFSO has ongoing efforts to compare and improve outcomes on an international level and developed a data dictionary set as the minimum to be reported in all bariatric registries. However, the outcomes presented in the IFSO global registry report 2019 show a lack of uniformity in gathered data points among contributing registries. One essential step in developing a CDE set is to assemble a task force [13, 18], such as the Registry Committee which has been commissioned to develop a core outcome set [8]. They have the ideal platform to facilitate, develop, share, and recommend using a CDE set that can be implemented internationally as the minimum set to be reported to encourage international collaborative investigations.

## Conclusion

There is only limited uniform agreement in variables across eleven of the 18 national bariatric surgery registries, emphasizing the ongoing inconsistency of reported outcomes and other characteristics in bariatric literature. Improving consistency by developing and implementing a common data element set in national registries will facilitate future international collaborative studies and international benchmarking.

Recommendations:

- Need for consistency in bariatric literature by reporting standardized outcomes using common data elements in national registries;
- International implementation of a common data element set in existing and developing national bariatric surgery registries for future nested registry trials, international collaborations, international benchmarking, and large population based studies; and
- Future work is needed for further alignment and uniformity in collected variables across registries with identical definition(s).

## Supplementary Information

ESM 1 (DOCX 21 kb)

## Declarations

### Disclaimer

The authors cannot take responsibility for variables misplaced due to the lack of a dictionary with specific definitions for variables.
