# Supplementary material for: National Bariatric Surgery Registries: an International Comparison
Source: Obes Surg. 2021 Mar 30;31(7):3031–9. doi: 10.1007/s11695-021-05359-0 (PMC8175300; doi:10.1007/s11695-021-05359-0)
Supplement: Supplementary file 1 — (DOCX 21 kb) [file 11695_2021_5359_MOESM1_ESM.docx]

**Supplementary table 1.** Summary of all the 250 variables divided into separate domains

|  | **Variables** |
| --- | --- |
| **Patient characteristics** | |
|  | Nationality |
|  | Patient ID no |
|  | Healthcare institution |
|  | Hospital ID |
|  | Initials |
|  | Prefix |
|  | Surname |
|  | Date of birth |
|  | Sex |
|  | Date of Death |
|  | Education status |
|  | Employment status |
|  | Phone number |
|  | Funding |
|  | Referral |
|  | Ethnicity |
| **Prior Bariatric History** | |
|  | Hospital ID |
|  | Healthcare institution of prior bariatric procedure |
|  | Prior existing comorbidities |
|  | Date of prior bariatric procedure |
|  | Prior metabolic or bariatric procedure |
|  | Prior type of gastric bypass |
|  | Prior type of malabsorptive procedure |
|  | Prior type of other bariatric procedure |
|  | Prior gastric band removal |
|  | Prior gastric band revision |
|  | Performed in bariatric healthcare centre |
|  | Performed in other healthcare centre |
|  | Prior gastric balloon |
|  | Date of prior comorbidities |
| **Screening** | |
|  | Healthcare Institution |
|  | Hospital ID |
|  | Date of consultancy |
|  | Height |
|  | Weight |
|  | Highest measured weight |
|  | Waist circumference |
|  | Body Mass Index (BMI) |
|  | Hypertension (diagnosis) |
|  | Diabetes mellitus (diagnosis) |
|  | Details diabetes mellitus |
|  | HbA1c (mmol/mol) |
|  | Dyslipidemia |
|  | LDL-cholesterol (mmol/L) |
|  | HDL-cholesterol (mmol/L) |
|  | Triglycerides (mmol/L) |
|  | LDL/HDL-ratio |
|  | GERD (diagnosis) |
|  | OSAS (diagnosis) |
|  | Osteoarthritis (diagnosis) |
|  | Obesity Surgery Mortality Risk Score (OS-MRS) |
|  | Myocardial infarction |
|  | Congestive heart failure |
|  | Peripheral vascular disease/ aneurysm aorta |
|  | CVA/TIA |
|  | Dementia |
|  | Chronic pulmonary disease |
|  | Gastrointestinal ulcer disease |
|  | Liver disease |
|  | Para-/hemiplegia |
|  | Renal disease |
|  | Malignancy (excluding cutaneous SCC, BCC) |
|  | HIV/AIDS |
|  | Connective tissue disease (including rheumatoid disease) |
|  | Asthma |
|  | Mobility |
|  | Increased risk Pulmonary Embolism |
|  | PCOS |
|  | Depression |
|  | Operation decision date |
|  | Renal transplant |
|  | Liver transplant |
|  | Referral date |
|  | Diabetes duration |
|  | Creatinine |
|  | PTH |
|  | Vitamin D status |
|  | Blood pressure (diastolic and systolic) |
|  | Diarrhea |
|  | Incontinence |
|  | Menstrual cycle |
|  | Panniculus |
|  | Other comorbidities |
|  | Smoking |
|  | Social activities |
|  | Previous weight loss program |
|  | Abdominal apronectomy |
|  | Pre-operative endoscopy |
|  | Abdominal ultrasound |
|  | Helicobacter pylori |
|  | pH measurement |
| **Operation** | |
|  | Healthcare Institution |
|  | Hospital ID |
|  | Preoperative Weight |
|  | ASA classification |
|  | Date of operation |
|  | Surgical procedure (primary/two-stage/revision) |
|  | Main reason revision |
|  | Type of revisional surgery (conversion/revision/undo) |
|  | Type of revisional bariatric procedure |
|  | Operative approach |
|  | Bariatric procedure |
|  | Surgeon ID |
|  | First assistant ID |
|  | Date of discharge |
|  | Type of technique gastric band |
|  | Dissection for band positioning |
|  | Fixation gastric band |
|  | Type malabsorptive |
|  | Type gastric bypass |
|  | Method measuring bowel length |
|  | Biliopancreatic limb length |
|  | Alimentary limb length |
|  | Closure Petersen’s space |
|  | Closure hernia jejuno-jejunostomy |
|  | Type gastric band (brand) |
|  | Circumference ring of gastric band |
|  | Common limb length |
|  | Bougie size |
|  | Technique of pouch excision |
|  | Other techniques for pouch excision |
|  | Distance from pylorus |
|  | Pouch size |
|  | Details of other operation(s) |
|  | Operation record status (incomplete/complete) |
|  | Age at operation |
|  | Aborted procedure |
|  | Planned or unplanned revision |
|  | Pre-operative Body Mass Index (BMI) |
|  | Type inserted instrument |
|  | Brand instrument |
|  | Model instrument |
|  | Producer instrument |
|  | Serial number instrument |
|  | Drainage |
|  | Provocative test for leakage |
|  | Time incision |
|  | Time end of procedure |
|  | Combined operation |
|  | Suture material |
|  | Ante-colic/retro-colic |
|  | Blood loss |
| **Complication** | |
|  | Healthcare Institution |
|  | Hospital ID |
|  | Date of complication |
|  | Period the complication occurred |
|  | Date of re-admission |
|  | Date of discharge after re-admission |
|  | Type of (re)intervention |
|  | Clavien Dindo classification of surgical complications |
|  | Operative approach (re)intervention |
|  | Anesthesia |
|  | ICU-admission |
|  | Date ICU admission |
|  | Date of discharge ICU |
|  | Patient status at discharge |
|  | Gastrointestinal perforation |
|  | Bleeding |
|  | Splenic injury |
|  | Liver injury |
|  | Source of bleeding |
|  | Surgical complications |
|  | Leak |
|  | Post-operative complications |
|  | Esophageal complications |
|  | Esophageal dilatation |
|  | Esophageal dysmotility |
|  | Gastric complication |
|  | Gastric ulcer |
|  | Marginal ulcer |
|  | Stricture |
|  | Delayed gastric emptying |
|  | Motility disorder |
|  | Metabolic disorder |
|  | Early dumping |
|  | Late dumping |
|  | Other deficiencies |
|  | Secondary hyperparathyroidism |
|  | Peripheral neuropathy |
|  | Electrolyte disorder |
|  | Hepatobiliary problems |
|  | Liver failure |
|  | CBD stones |
|  | Band problems |
|  | Pouch dilatation/band slippage |
|  | Band erosion |
|  | Port/band infection |
|  | Motility disorder due to gastric band |
|  | Other complications (including cardiac, pulmonary and other) |
|  | Incisional hernia |
|  | Intestinal obstruction |
|  | Intolerance of bariatric procedure |
|  | Petersen’s hernia |
|  | Extended admission |
|  | Malnutrition/enteral feeding |
|  | Post-op vomiting/nausea |
|  | Other procedure related complications |
|  | Hospital discharge destination (home/revalidation centre) |
|  | Acute renal failure |
| **Follow-up** | |
|  | Healthcare institution |
|  | Hospital ID |
|  | Date of follow-up |
|  | Weight |
|  | Hypertension status |
|  | Medical treatment hypertension |
|  | Diabetes mellitus status |
|  | HbA1c (mmol/mol) |
|  | Medical treatment diabetes mellitus |
|  | Dyslipidemia status |
|  | Medical treatment dyslipidemia |
|  | GERD status |
|  | Medical treatment GERD |
|  | OSAS status |
|  | Medical treatment OSAS |
|  | Osteoarthritis |
|  | Medical treatment osteoarthritis |
|  | Diarrhea |
|  | Depression |
|  | Abdominal pain for no clinical reason |
|  | How was follow-up conducted (hospital or by phone) |
|  | Blood tests |
|  | Clinical malnutrition |
|  | Education status |
|  | Asthma |
|  | Mobility |
|  | PCOS |
|  | Urinary incontinence |
|  | Menstrual function |
|  | Pregnancy |
|  | Estimated date of delivery |
|  | Follow-up record status |
|  | Follow-up period |
|  | Patient called for follow-up |
|  | Contact details patient |
|  | Lost to follow-up |
|  | Lost to follow-up date |
|  | Self-reported weight |
|  | Body Mass Index (BMI) |
|  | Excess weight |
|  | Ideal weight |
|  | Lost weight |
|  | Excess Weight Loss % |
|  | Total Weight Loss % |
|  | Email to surgeon |
|  | Age at primary operation (in case unknown at primary registration) |
|  | Hunger feeling |
|  | Vitamins and microelements intake |
|  | Abdominal apron |
|  | Endoscopy |
|  | Abdominal ultrasound |

Abbreviations: ID, Identity Document; BMI, Body Mass Index; HbA1c, Hemoglobin A1c; LDL, Low-Density Lipoprotein; HDL, High-Density Lipoprotein; GERD, Gastroesophageal Reflux Disease; OSAS, Obstructive Sleep Apnea Syndrome; CVA, Cerebrovascular Accident; TIA, Transient Ischemic Attack; SCC, Squamous Cell Carcinoma; BCC, Basal Cell Carcinoma; HIV, Human Immunodeficiency Virus; AIDS, Acquired Immune Deficiency Syndrome; PCOS, Polycystic Ovary Syndrome; PTH, Parathyroid Hormone; pH, Pondus Hydrogenii; ASA, American society of Anesthesiologists; ICU, Intensive Care Unit; CBD stones, common bile duct stones.

Adolescent section of the NBSR and the pre-operative work-up section of OGA are not included in the list of common data elements due to registration specific variables.
